# Supplementary material for: Theileria parasites sequester host eIF5A to escape elimination by host-mediated autophagy
Source: Nat Commun. 2024 Mar 12;15:2235. doi: 10.1038/s41467-024-45022-7 (PMC10933305; doi:10.1038/s41467-024-45022-7)
Supplement: Supplementary file 1 — Supplementary Information [file 41467_2024_45022_MOESM1_ESM.pdf]

SUPPLEMENTARY INFORMATION

TITLE

Theileria parasites sequester host eIF5A to escape elimination by host-mediated autophagy

AUTHORS

Marie Villares[1], Nelly Lourenço[1], Ivan Ktorza[1], Jeremy Berthelet[1], Aristeidis Panagiotou[1], Aurélie Richard[1], Angélique Amo[1], Yullianna Koziy[1], Souhila Medjkane[1], Sergio Valente[2], Rossella Fioravanti[2], Catherine Durieu[3], Laurent Lignière[3], Guillaume Chevreux[3], Antonello Mai[2,4], Jonathan B. Weitzman[1]

AFFILIATIONS

1 Université Paris Cité, CNRS, UMR7126 Epigenetics and Cell Fate, 75013 Paris, France

2 Department of Drug Chemistry & Technologies, Sapienza University of Rome, 00185 Rome, Italy

3 Université Paris Cité, CNRS, UMR 7592 Institut Jacques Monod, 75013 Paris, France

4 Pasteur Institute, Cenci-Bolognetti Foundation, Sapienza University of Rome, 00185 Rome, Italy

a

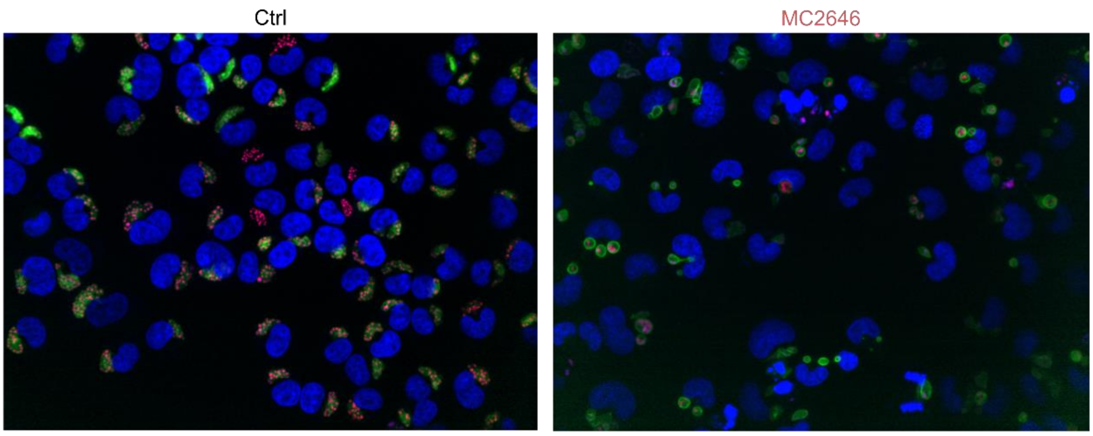
Ctrl
MC2646

b

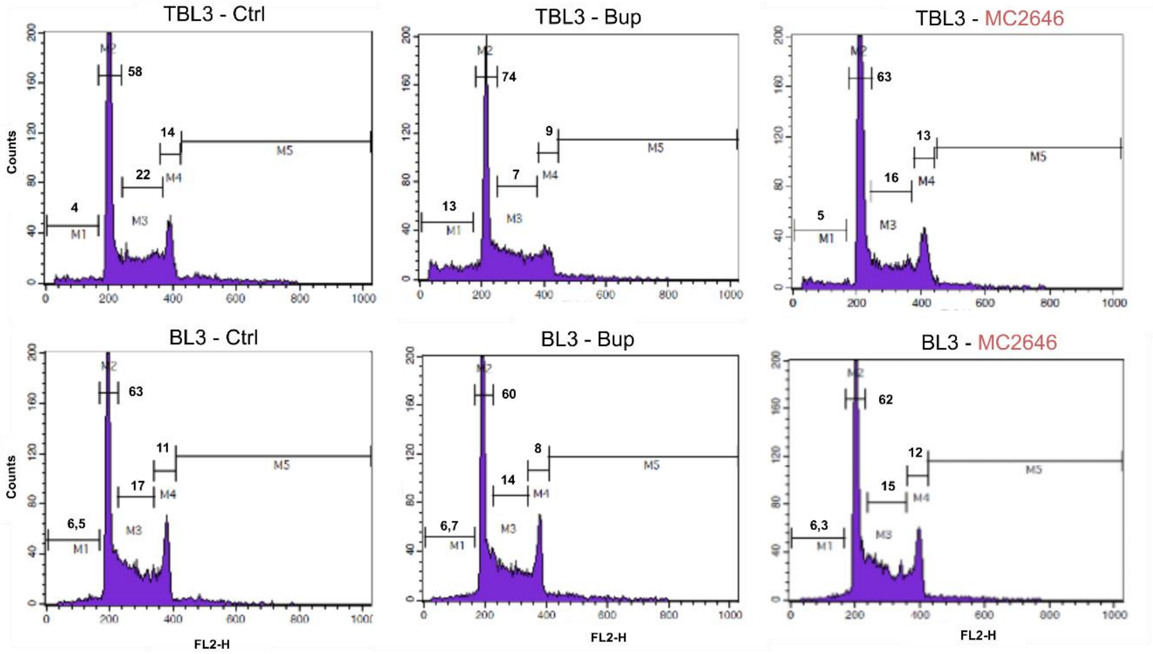
TBL3 - Ctrl
TBL3 - Bup
TBL3 - MC2646
BL3 - Ctrl
BL3 - Bup
BL3 - MC2646
Counts
FL2-H

c

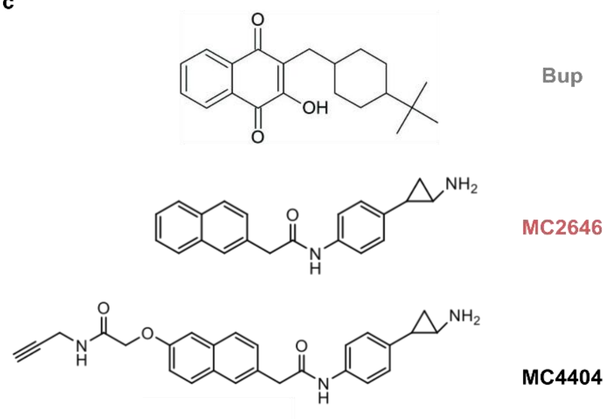
Bup
MC2646
MC4404

Supplementary Figure 1

a) Examples of images from microscopy-based drug screen, as previously described[25]. *T. annulata*-infected macrophages (Tac12 cell line) were distributed into 96-well plates and imaged after incubation (48 h) with candidate drug targets at 10 µM. The screen involved detection of host and parasite cell nuclei (DAPI staining, blue), the macroschizont membrane (CLASP-GFP fusion protein, green) and parasite nuclei (H3K18me1 staining, red). The left image shows a typical field (30 fields sampled) analysed by the Opera Phenix microscope (Perkin Elmer, Photonic BioImaging platform, Pasteur Institute) and the associated Acapella Software to monitor host and parasite survival. The right image shows a typical field treated with MC2646 compound. Note the shrunken schizont structure in green and reduced parasite nuclei per cell (red).

b) Cell cycle analysis by flow cytometry performed on BL3 and TBL3 cells, either untreated or incubated with Buparvaquone (Bup) or MC2646 (1 µM for 48 h). The percentage of dying cells is indicated as the sub-G1 (M1) population.

c) Chemical structures of the Buparvaquone and MC2646 compounds and the MC4404 'click' derivate of MC2646.

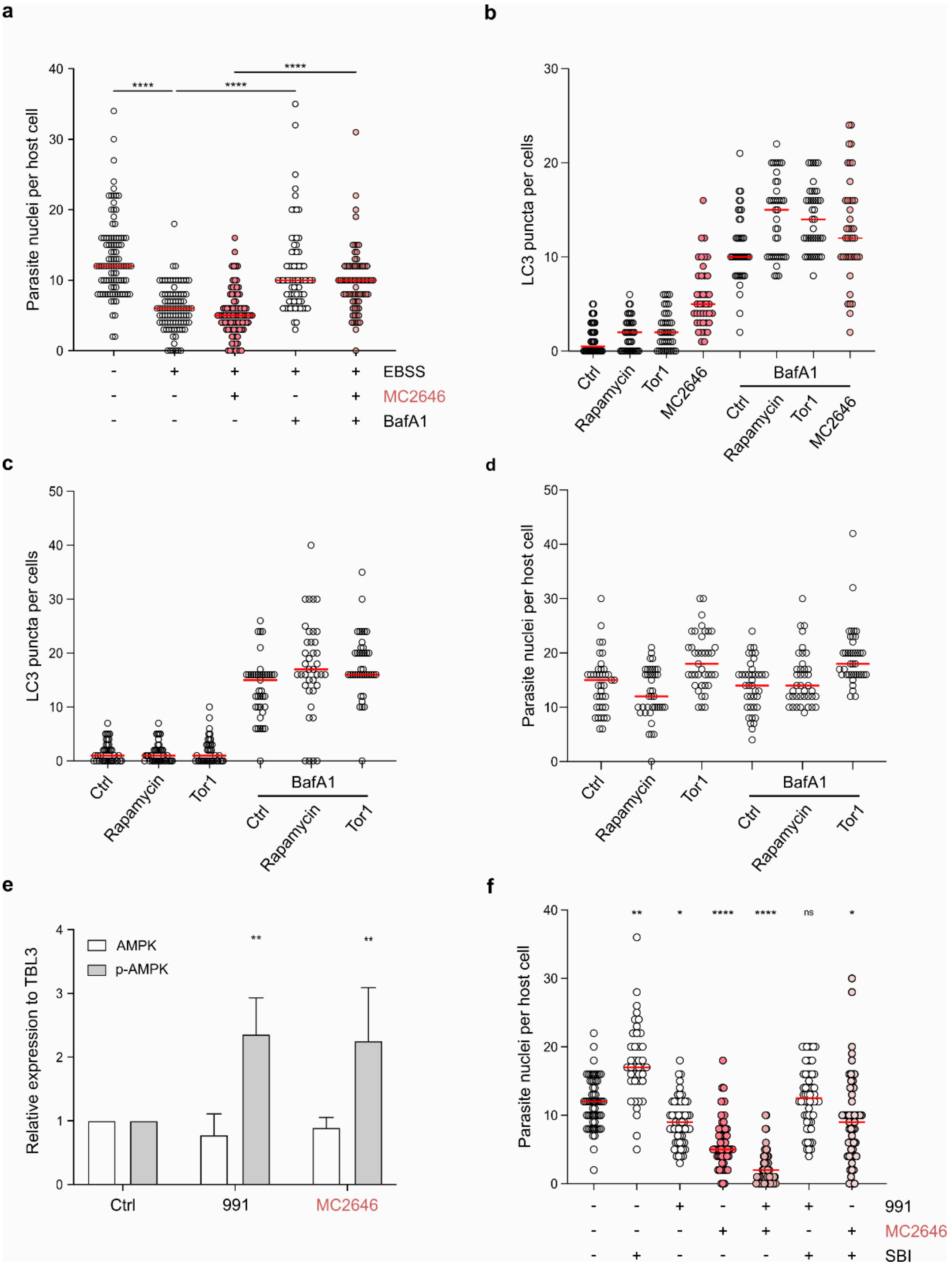

Supplementary Figure 2

a) The effect of EBSS and MC2646 treatment on parasite survival in Tac12 infected macrophages. The experimental conditions are the same as those used for TBL3 cells in Figure 2e. The parasite load was measured by parasite nuclei per host macrophage cell. At least 50 cells were quantified for each condition. Results are significant under Kruskal-Wallis followed by a Dunn's multiple comparison test ****$p<0.0001$.

b) Regulators of autophagy were tested in uninfected BL3 cells. Cells were incubated with Rapamycin (Rap, 2 µM), Torin1 (Tor1, 1 µM) or MC2646 with or without Bafilomycin (BafA1, 1 µM) for 24 hours. The formation of LC3B puncta was monitored per cell by immunofluorescence. This experiment demonstrated the effective induction of autophagy by Rapamycin or Torin1 in uninfected cells.

c) Effect of treatment with regulators of autophagy in infected TBL3 cells. TBL3 cells were incubated with Rapamycin (Rap, 2 µM), Torin1 (Tor1, 1 µM) with or without Bafilomycin (BafA1, 1 µM) for 24 hours. LC3B puncta formation was monitored per cell by immunofluorescence. This experiment demonstrated that the effects of these regulators of autophagy were blocked in infected TBM3 cells.

d) Quantification of parasite load upon Rapamycin or Torin1 of infected TBL3 cells. Cells were treated with Rapamycin (Rap, 2 µM), Torin1 (Tor1, 1 µM) with or without Bafilomycin (BafA1, 1 µM) for 24 hours. Parasite nuclei per host cell were monitored by counting DAPI-stained images (at least 50 cells per condition). No statistically significant changes were observed. These results suggest that the mTOR pathway is not implicated in parasite survival in these cells.

e) Quantification of the Western blot analysis of AMPK and p-AMPK levels, normalized by Ponceau, of the triplicate experiments show in Figure 2c. Statistical significance 2-way Anova Sidak's multiple comparisons over TBL3 Control (=untreated, Ctrl) (n=3 ; ** $p < 0.01$).

f) Treatment of TBL3 cells with an inhibitor of the AMPK pathway (SBI) rescued the impact of MC2646 on parasite survival. Parasite nuclei per host cell were monitored upon treatment with SBI, MC2646 or 991 drugs. Results are significant under Kruskal-Wallis followed by a Dunn's multiple comparison test **** $p<0.0001$.

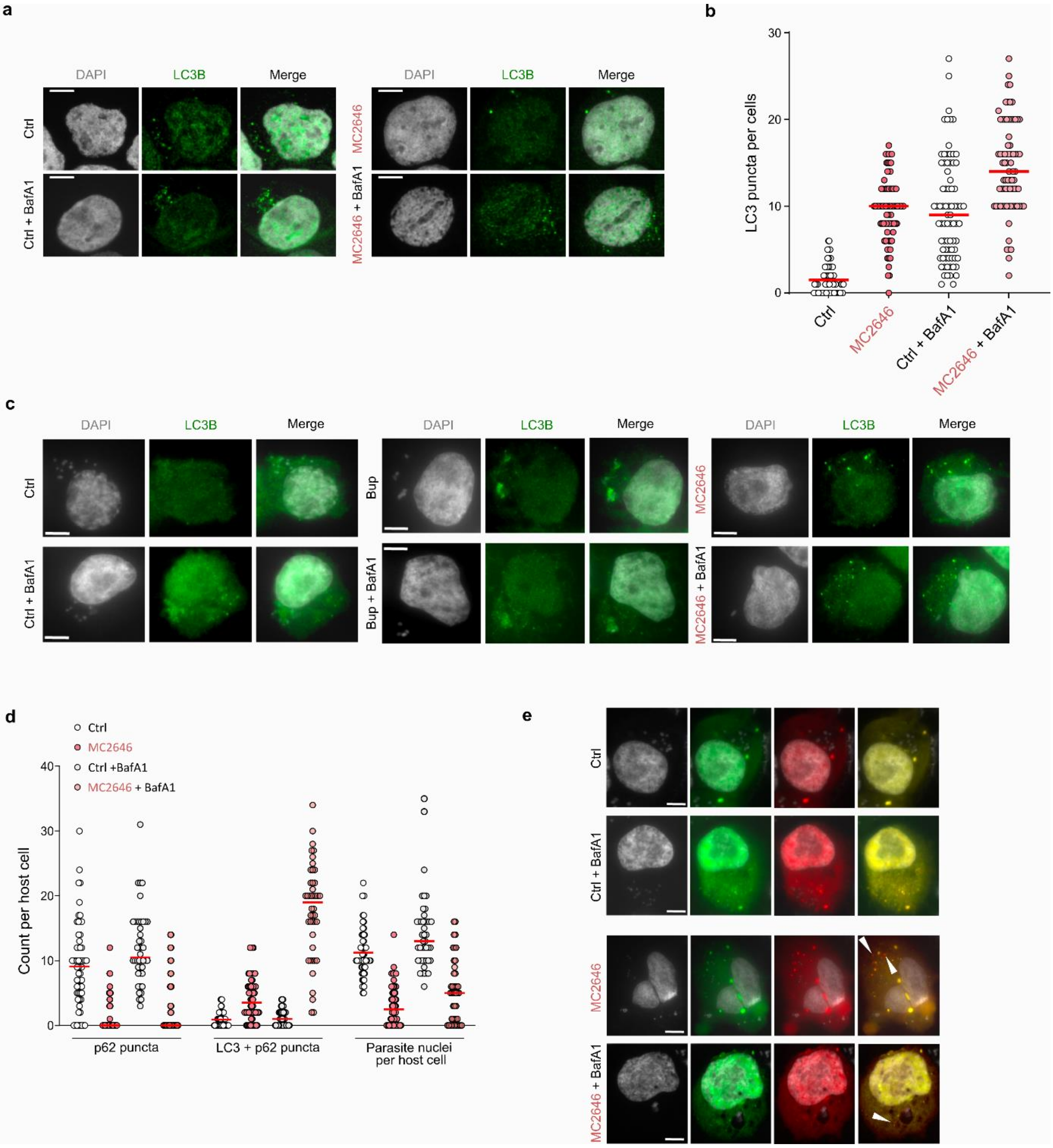

Supplementary Figure 3

a) Immunofluorescence analysis of uninfected BL3 cells treated or not with MC2646 for 24 h. BafilomycinA1 (BafA1) was added (50 nM for 3 h). LC3B puncta formation was monitored by immunofluorescence.

b) Quantification of the formation of LC3 puncta in TBL3 cells treated with MC2646 for 24 h, with or without BafilomycinA1 (BafA1) addition (50 nM for 3 h). The puncta quantification is the average of 3 independent experiments. Results are significant under Kruskal-Wallis followed by a Dunn's multiple comparison test ****$p<0.0001$.

c) Immunofluorescence analysis of infected TBL3 cells treated or not with Buparvaquone (Bup) or MC2646 for 24 h. BafilomycinA1 (BafA1) was added (50 nM for 3 h). This is a representative experiment of the 3 that are shown quantitatively in Figure 3d.

d) Quantification of p62 puncta alone or p62-LC3B puncta (autophagosomes) in control (Ctrl) condition or treated with MC2646, in the presence or absence of BafA1. MC2646 increases colocalization between LC3 and p62, which was enhanced when the autophagic flux was blocked. The increase in autophagosome number was anti-correlated with parasite nuclei number per host cells.

e) Infected TBL3 cells were transfected with a LC3-RFP-GFP reporter plasmid and treated with MC2646 and/or BafA1. We highlight the red spots (white arrowheads) , indicating the activation of autophagic flux, observed upon MC2646 drug treatment and enhanced when combined with BafA1. The localization of the parasite nuclei is indicated by arrows.

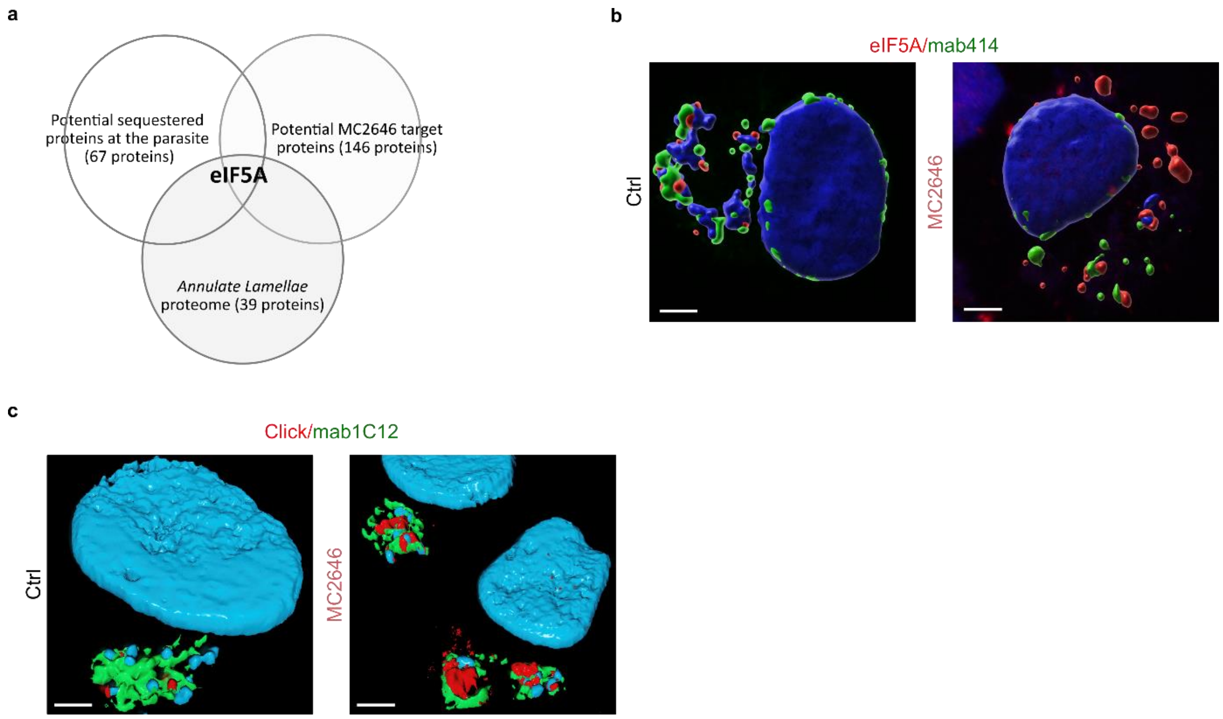

Supplementary Figure 4

a) Schematic representation of the cross-analysis of proteome data from MC2646 Pull-down/Mass Spec, published BioID-MS data of host protein sequestered by the parasite[16] and proteins enriched in annulate lamellae[38].

b) Reconstructed 3D microscopy images of infected TBL3 cells stained with mab414 to mark the annulate lamellae structures (green) and a specific anti-eIF5A antibody (red) in cells treated or not with MC2646 compound. The parasite and host nuclei are marked by DAPI staining (blue).

c) Immunofluorescence analysis showing that TBL3 cells treated with Buparvaquone still show a parasite localization of eIF5A (green) and MC2646 (red).

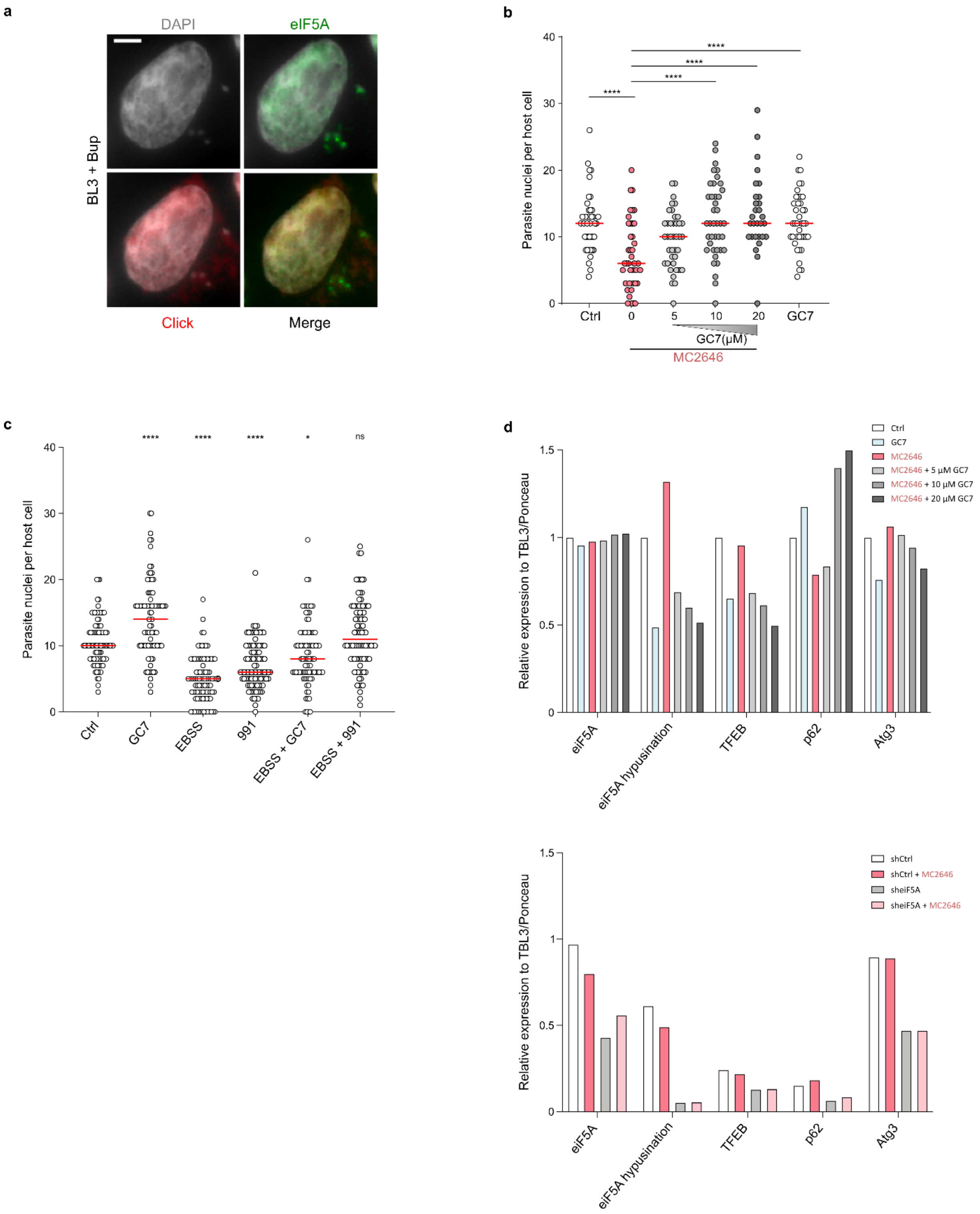

Supplementary Figure 5

a) Buparvaquone treatment does not lead to eIF5A release. Infected TBL3 cells were incubated with Buparvquone (Bup) for 24 hours and then the localization of the host eIF5A proteins was monitored by immunofluorescence (green) and the 'click' MC4404 compound (red)

b) Inhibition of eIF5A hypusination rescued parasite survival. Infected Tac12 macrophages were subjected to the treatment conditions (Figure 4c-d, with or without MC2646 and increasing concentrations of the DHPS inhibitor GC7) and monitored for parasite load, as measured by parasite nuclei per host cell. n=3

c) The effect of hypusination inhibitor GC7 on parasite elimination induced by the autophagy inducers EBSS or 991 compound. Infected TBL3 cells were incubated in EBSS media (4h) or the 991 AMK1 activator (4 µM) in the presence of absence of GC7 and parasite load (determined by number of nuclei per host cell) was monitored.

d) Quantification of the Western blot analysis results presented in Figure 4e and 4h.

In all experiments, cells were incubated with 50 ng/ml Buparvaquone or 4 µM MC2646 for 24h and/or BafA1 (50 nM for 3h). Results are representative of 3 independent experiments. Statistical analysis Dunnett's multiple comparison test; ****$p<0.0001$.

**qPCR Gene :**	**Foward**	**Reverse**
TamR1	CCACTCCTGTAGCGGGTAAA	TTGGGAGGTACTGACCCAAA
Hsp70	ACGCAAATGGAATCCTCAAC	TATTCGTCGTGCTCTGCTAA
**shCtrl :**	Empty vector - pLKO gfp Addgene #30323	
**siRNA:**		
siEIF5A_1(+) [53 nt] : CCGG gcaaggagattgagcagaa CTCGAG ttctgctcaatctccttgc TTTTT		
siEIF5A_1(-) [53 nt] : AATTAAAAA gcaaggagattgagcagaa CTCGAG ttctgctcaatctccttgc		

Supplementary Table 1

Antibodies used	Reference	Supplier	Species	Dilution used
LC3B	ab51520	Abcam	Rabbit	1/250 IF - 1/2000 Western
AMPK	#2532S	Cell Signaling	Rabbit	1/200 Western
AMPK-p	#2531S	Cell Signaling	Rabbit	1/200 Western
TFEB	#13372-1-AP	Proteintech	Rabbit	1/300 IF - 1/2000 Western
eIF5A	#611976	BD Biosciences	Mouse	1/10000 Western
eIF5A	ab137561	Abcam	Rabbit	1/200 IF
eIF5A hypusinated	ABS1064-I	EMD Millipore	Rabbit	1/4000 Western
Atg3	ab108251	Abcam	Rabbit	1/ Western
p62/SQSTM1	ab56416	Abcam	Mouse	1/ Western
mab414	ab24609	Abcam	Mouse	1/500 IF
Actin	A1978	Sigma	Mouse	1/10000 Western

Supplementary Table 2
