# Supplementary material for: Theileria parasites sequester host eIF5A to escape elimination by host-mediated autophagy
Source: Nat Commun. 2024 Mar 12;15:2235. doi: 10.1038/s41467-024-45022-7 (PMC10933305; doi:10.1038/s41467-024-45022-7)
Supplement: Supplementary file 3 — Description of Additional Supplementary Files [file 41467_2024_45022_MOESM3_ESM.pdf]

Description of Additional Supplementary Files

File Name: Supplementary Data 1
Description: Full mass spectrometry data
