# Supplementary material for: Theileria parasites sequester host eIF5A to escape elimination by host-mediated autophagy
Source: Nat Commun. 2024 Mar 12;15:2235. doi: 10.1038/s41467-024-45022-7 (PMC10933305; doi:10.1038/s41467-024-45022-7)
Supplement: Supplementary file 5 — Reporting Summary [file 41467_2024_45022_MOESM5_ESM.pdf]

nature portfolio

Corresponding author(s): Jonathan B WEITZMAN

Last updated by author(s): Dec 19, 2023

Reporting Summary

Nature Portfolio wishes to improve the reproducibility of the work that we publish. This form provides structure for consistency and transparency in reporting. For further information on Nature Portfolio policies, see our Editorial Policies and the Editorial Policy Checklist.

Statistics

For all statistical analyses, confirm that the following items are present in the figure legend, table legend, main text, or Methods section.

n/a	Confirmed	
☐	☒	The exact sample size (*n*) for each experimental group/condition, given as a discrete number and unit of measurement
☐	☒	A statement on whether measurements were taken from distinct samples or whether the same sample was measured repeatedly
☐	☒	The statistical test(s) used AND whether they are one- or two-sided<br>*Only common tests should be described solely by name; describe more complex techniques in the Methods section.*
☐	☒	A description of all covariates tested
☒	☐	A description of any assumptions or corrections, such as tests of normality and adjustment for multiple comparisons
☐	☒	A full description of the statistical parameters including central tendency (e.g. means) or other basic estimates (e.g. regression coefficient) AND variation (e.g. standard deviation) or associated estimates of uncertainty (e.g. confidence intervals)
☐	☒	For null hypothesis testing, the test statistic (e.g. *F*, *t*, *r*) with confidence intervals, effect sizes, degrees of freedom and *P* value noted<br>*Give P values as exact values whenever suitable.*
☒	☐	For Bayesian analysis, information on the choice of priors and Markov chain Monte Carlo settings
☒	☐	For hierarchical and complex designs, identification of the appropriate level for tests and full reporting of outcomes
☒	☐	Estimates of effect sizes (e.g. Cohen's *d*, Pearson's *r*), indicating how they were calculated

Our web collection on statistics for biologists contains articles on many of the points above.

Software and code

Policy information about availability of computer code

Data collection	No software were used for data collection.
Data analysis	For RNA-Seq analysis<br>The RNA-Seq data were analysed on the cluster of French Institute of Bioinformatics (IFB), using the workflow developed by the BiBs platform (version 0.5) and based on RASflow49, which integrates all the following steps. Trimming of adapters and low-quality reads was done by Trim Galore! and we used the HISAT2 aligner50 to map the reads on the bovine genome (assembly accession GCF_002263795.1 for Bos Taurus). The resulting mapped reads were assigned to genomic features ("gene" parameter) using featureCounts on a similarly fused GTF annotation file. Finally, the differential expression analysis (DEA) was carried out using DESeq251. The two datasets were analyzed separately. The exact configurations for the workflow can be found attached. Gene set enrichment analysis (GSEA)52 was carried out using the version v4.2.3. windows application. The gene counts produced by RASflow were used as input for the GSEA of each dataset. The geneset database was created by subsetting MSigDB v7.4, for autophagy-related genesets. The Bovine.chip file, as well as the parameters for each comparison (.rpt) can be found attached.

For manuscripts utilizing custom algorithms or software that are central to the research but not yet described in published literature, software must be made available to editors and reviewers. We strongly encourage code deposition in a community repository (e.g. GitHub). See the Nature Portfolio guidelines for submitting code & software for further information.

Data

Policy information about availability of data

All manuscripts must include a data availability statement. This statement should provide the following information, where applicable:

- Accession codes, unique identifiers, or web links for publicly available datasets
- A description of any restrictions on data availability
- For clinical datasets or third party data, please ensure that the statement adheres to our policy

All deposited data and accession numbers will be provided upon acceptance

Research involving human participants, their data, or biological material

Policy information about studies with human participants or human data. See also policy information about sex, gender (identity/presentation), and sexual orientation and race, ethnicity and racism.

Reporting on sex and gender	n/a
Reporting on race, ethnicity, or other socially relevant groupings	n/a
Population characteristics	n/a
Recruitment	n/a
Ethics oversight	n/a

Note that full information on the approval of the study protocol must also be provided in the manuscript.

Field-specific reporting

Please select the one below that is the best fit for your research. If you are not sure, read the appropriate sections before making your selection.

☒ Life sciences ☐ Behavioural & social sciences ☐ Ecological, evolutionary & environmental sciences

For a reference copy of the document with all sections, see nature.com/documents/nr-reporting-summary-flat.pdf

Life sciences study design

All studies must disclose on these points even when the disclosure is negative.

Sample size	Sample size was determined to be adequate based on the magnitude and consistency of measurable differences between groups. Sample size decisions were based on the standards used in publications in the field. For all microscopy quantification, we sampled over 50 cells and performed all experiments in triplicate. These criteria were based on previous published studies Cheeseman et al. Nature Communications 2021 PMID: 34050145 and Villares et al. Communications Biology 2022 PMID: 36380082. We chose >30 cells to analyse in order to ensure that we could do parametric test (either ANOVA or t-test) every time it is needed.
Data exclusions	Data were only excluded for failed experiments.
Replication	All experiments were replicated at distinct dates at least three times. Replicate experiments were successful.
Randomization	After discussion with colleagues it was decided that randomization approaches were not relevant for this study.
Blinding	We considered that blinding was not relevant for most of these experiments based on data reproducibility. An exception was the quantification of induction of merogony experiments, in which two independent researchers performed quantification under blinded conditions. In the initial stages of this study, several blinded experiments were performed and quantitated manually by different members of the team. We did not obeserve significant differences between experimators in these studies.

Reporting for specific materials, systems and methods

We require information from authors about some types of materials, experimental systems and methods used in many studies. Here, indicate whether each material, system or method listed is relevant to your study. If you are not sure if a list item applies to your research, read the appropriate section before selecting a response.

Materials & experimental systems

n/a	Involved in the study
☐	☒ Antibodies
☐	☒ Eukaryotic cell lines
☒	☐ Palaeontology and archaeology
☒	☐ Animals and other organisms
☒	☐ Clinical data
☒	☐ Dual use research of concern
☒	☐ Plants

Methods

n/a	Involved in the study
☒	☐ ChIP-seq
☒	☐ Flow cytometry
☒	☐ MRI-based neuroimaging

Antibodies

Antibodies used

We used these antibodies:
Antibodies for Western blot analysis
eIF5A (BD Biosciences, ref611976, 1/10000)
eIF5A-hypusinated (EMD Millipore Corp, ABS1064, 1/4000)
AMPK (Cell Signaling, 2532S, 1/2000)
755 pAMPK (Cell Signaling, 2531S, 1/2000)
LC3B (Abcam, ab51520, 1/2000)
p62 (Abcam, ab56416, 1/2500)
ATG3 (Abcam, ab108251, 1/2000)
TFEB (Proteintech, ref 13372, 1/2000)
Actin (Sigma, A1978,1/10000)

Antibodies for Immunofluorescence and Microscopy
eIF5A (Abcam, ab137561, 1/200)
TFEB (1/300); p62 (Abcam, ab56416, 1/500)
LC3B (Proteintech, ref 18725, 1/250)
mab414 (Abcam, 24609, 1/500).
rabbit anti-H3K18me1 (ab177253 Abcam) 1/5000;
Secondary Antibody 1:1000 dilution (Invitrogen).

anti-6xHis-tag (SAB270221, Sigma), 1/10000
anti-mouse HRP-secondary (G-2104, Thermofisher), 1/50000
anti-rabbit HRP-secondary (31460, Thermofisher), 1/50000

Validation

Validation can be found on the manufacturers' technical sheets with specific references.

Eukaryotic cell lines

Policy information about cell lines and Sex and Gender in Research

Cell line source(s)

All infected bovine cell lines used in this study were previously described: TBL3 cells were derived from in vitro infection of the spontaneous bovine-B lymphosarcoma cell line, BL3, with Hissar stock of T.annulata. The TpMD409 lymphocyte cell line are infected with T. parva. The TaC12 is a T. annulata infected bovine macrophage cell line. All parasite-infected cell lines were provided by colleagues in the field as indicated in the text. We acknowledged G. Langsley (Institut Cochin, Paris, France) for the TBL3, BL3 and TpMD409 cell lines and K. Woods (University of Bern, Switzerland) for Tac12 cell line in the text. These cell lines have been previously used an reported by several labs including ours (see Villares et al. Communications Biology 2022 PMID: 36380082, Cheeseman et al. Nature Communications 2021 PMID: 34050145). The U2OS-LC3-RFP/GFP cell line was obtained from Guido Kroemer, Université Paris Cité.

Authentication

The cells were authenticated as infected by Theileria parasites with PCR of several parasite genes.

Mycoplasma contamination

All cells lines were tested (negatively) for mycoplasma on a monthly basis

Commonly misidentified lines
(See ICLAC register)

No cell lines used are listed in the database of commonly misidentified cell lines.
